# The prevalence of malnutrition among critically ill children: a systematic review and meta-analysis

**DOI:** 10.1186/s12887-023-04419-x

**Published:** 2023-11-21

**Authors:** Eyob Girma Abera, Habtamu Sime

**Affiliations:** 1 Department of Public Health, Jimma University, P.O.Box 378, Jimma, Oromia Ethiopia; 2 Clinical Trial Unit, Jimma University, Jimma, Oromia Ethiopia; 3 Department of Pediatrics, Jimma University, Jimma, Oromia Ethiopia

**Keywords:** Malnutrition, Critically ill, Children, Systematic review, meta-analysis

## Abstract

**Background:**

Critically ill children have a lower nutritional reserve, compounding the restricted food intake during intensive care unit (ICU) and hospital stays, and scarce data are available to point out the problem. Therefore, this review aimed to assess the pooled prevalence of malnutrition among critically ill children.

**Methodology:**

This systematic review was conducted in accordance with the JBI methodology for systematic reviews of prevalence and incidence. Databases including, PubMed/MEDLINE, CINAHL/EBSCO, HINARI, Google Scholar, and gray literatures were used to find relevant articles. Eligible studies were critically appraised by two independent reviewers. Systematic review and meta-analysis was conducted using STATA 17 software. Funnel plot and at the 5% significance level, Egger’s test were used to check for publication bias.

**Result:**

From a total of 15 studies with 4331 study participants, the pooled prevalence of malnutrition in critically ill children was 37.19% (95% CI; 35.89–38.49) with a significant statistical heterogeneity (I^2^ = 98.6, *P* = < 0.0001). High income countries reported the lower pooled prevalence of malnutrition among critically ill children (30.14%, 95% CI; 28.41, 31.88). No publication bias was reported and sensitivity analysis suggested that no significance difference was shown in the prevalence of malnutrition among critically ill children with the pooled prevalence.

**Conclusion:**

The current systematic review and meta-analysis showed that more than one in three critically ill children was malnourished. Serious medical conditions in children that deserve admission to the intensive care unit could be a complication of malnutrition that may end up in deaths unless the undernutrition is addressed together with critical care intervention. Hence, specific strategies to prevent malnutrition among this neglected segment should be integrated with the existing healthcare systems and nutritional programs.

**Supplementary Information:**

The online version contains supplementary material available at 10.1186/s12887-023-04419-x.

## Introduction

The most significant developmental milestone changes during childhood are subject to higher nutritional demands [1]. However, malnutrition among children is a major public health problem, particularly in low and middle-income countries (LMICs). In 2020, the prevalence of malnutrition under-five were 28.7% while nearly half of all deaths in children under 5 are attributable to undernutrition [2]. The consequence of malnutrition is diverse and associated with longer negative health consequences such as developmental delay, increased susceptibility to infections, neurocognitive problems, and generational defects [3].

Neuroendocrine, metabolic, and immunologic changes are brought on by the metabolic response to acute circumstances such as trauma, infection, or surgery in critical illness [4]. Pediatric intensive care unit (PICU) admission rates for undernutrition in critically sick children range from 8.1 to 71.7%, despite variations in nutritional indices, the presence of chronic illness, age, and critical illness severity [5]. In light of this, early detection of juvenile undernutrition and the observation of nutritional status degradation might result in fast and appropriate nutritional therapies, which may improve clinical outcomes. Additionally, because most critically sick patients have reduced nutritional reserves, iatrogenic underfeeding and increasing malnutrition are further encouraged by prolonged fasting and frequent feeding pauses during intensive care unit (ICU) and hospital stays [6].

In Ethiopia, an institution-based prospective observational study was conducted at the University of Gondar Comprehensive Specialized Hospital for 18 months, and of the total admitted children to ICU, 47.97% were undernourished, of which 32% (95%CI: (26.8–37.4%) were severely wasted [1]. Similarly, a retrospective cross sectional study was conducted on 243 children to assess the prevalence malnutrition in critically ill children, and the overall prevalence of malnutrition was 83.7% [7]. Despite numerous studies that have been conducted on pediatric malnutrition, systematically reviewed data on the prevalence of malnutrition among critically ill children has not been published so far. Therefore, this systematic review and meta-analysis aimed to assess the prevalence of malnutrition among critically ill children, which will be used by nutrition program implementers, policymakers, and concerned stakeholders to achieve sustainable development goals.

## Methods

This systematic review was conducted in accordance with the JBI methodology for systematic reviews of prevalence and incidence [8] with the updated guideline of the Preferred Reporting Items for Systematic Reviews and Meta-Analysis (PRISMA 2020) [9] (Supplemental Table 1).

### Eligibility criteria

The inclusion criteria for this review and meta-analysis were: all critically ill children with an age group of under 18 years old; studies that had been conducted and reported in English; articles with a publicly available full text; and observational studies (cross-sectional, case-control, cohort studies, and longitudinal studies). However, studies that did not define the age of participants (above 18-year-old age group), studies without publicly available full-text, studies in languages other than English, studies that duplicated published literature, and studies that could not extract important outcome data were excluded.

### Search strategy

The search strategy aimed to locate both published and unpublished studies. The international databases PubMed/MEDLINE, CINAHL/EBSCO, HINARI, and Google Scholar were accessed to find relevant articles. A Medical Subject Headings (MeSH), keyword terms and phrases were used both in separation and in combination using the Boolean operators “OR” and “AND” to search for eligible articles. The reference list of all included sources of evidence was screened for additional studies. The task of searching sources was carried out from all stated electronic databases performed during August 29–31, 2022.

With the MeSH terms, “Malnutrition” and “Critical Illness*”, the keywords and phrases: “Malnutrition”, “Nutritional Deficienc*, “Under nutrition”, “Under-nutrition”, “Undernutrition”, “Wasting”, “Malnourishment”, “undernourishment”, “Malnourished”, “Nutrition deficienc*”, “Hypo nutrition*”, “Nutritional disorders*”, “Critical Illness*”, “Illness, Critical”, “Illnesses, Critical”, “Critically Ill”, “Intensive Care”, “Care, Critical”, “ICU Patient*”, “Care, Intensive”, “ICU Intensive Care”, “children”, “pediatrics”, ‘‘preschool children’’, prevalence, incidence, magnitude, burden, and proportion were used in separation or in combination to retrieve relevant articles on malnutrition in critically ill children.

### Study selection

Two impartial reviewers looked over the titles and abstracts before comparing them to the inclusion criteria. The JBI System for the Unified Management, Assessment and Review of Information (JBI SUMARI) (JBI, Adelaide, Australia) was used to extract potentially pertinent papers in their entirety and import their citation information [10]. Two independent reviewers thoroughly evaluated the complete text of the chosen citations in relation to the inclusion criteria. Discussion was used to settle any discrepancies that arose between the reviewers at each level of the selection process.

### Outcome measurements

The primary outcome of this systematic review and meta-analysis is the pooled prevalence of malnutrition among critically ill children. Studies that used anthropometric measurements (height-for-age, weight-for-height, weight-for-age) to assess malnutrition in critically ill children were included. Thus, stunting - height-for-age <-2 SD of the WHO Child growth standards median; wasting - weight-for-height <-2 SD of the WHO Child growth standards median; and underweight - weight-for-age <-2 standard deviations (SD) of the WHO Child growth standards median [11].

### Outcome operational definition

The term malnutrition refers to any type of alteration of the nutritional status and includes nutritional deficiencies, obesity, and the use of inappropriate diets. However, in this article, the term is typically used to refer to nutritional deficiencies (undernutritions).

### Assessment of methodological quality

Eligible studies were critically appraised by two independent reviewers for methodological quality in the review using standardized critical appraisal instruments from JBI for observational studies [12]. Any disagreements that arose during appraisal were resolved through discussion. The results of the critical appraisal were reported in narrative form and in a table. All studies that met a certain quality threshold underwent data extraction and synthesis.

### Data extraction

Data were extracted from studies included in the review by two independent reviewers using the standardized data extraction tool for prevalence and incidence available in JBI SUMARI [12]. The extracted data includes specific details about the condition, populations, study methods, measured outcome, and description of the main result. Any disagreements that arose between the reviewers were resolved through discussion.

### Publication bias and heterogeneity

Heterogeneity was assessed statistically using the standard chi-squared and I squared tests. When I^2^ exceeds 75%, high heterogeneity was declared [13] and a subgroup analysis was conducted to manage heterogeneity among studies using measurements including income level and study design. The possible risk of publication bias was examined by inspection of the funnel plot and statistically using Egger’s regression test. Besides, a sensitivity analysis was performed to examine the influence of a single study on the overall estimate.

### Data synthesis

The extracted data were pooled in a statistical proportional meta-analysis using JBI SUMARI. Effect sizes were expressed as a proportion with 95% confidence intervals around the summary estimate. The adjusted odds ratio (AOR) with its upper and lower bounds was extracted for significant variables. Statistical analyses were performed using the Freeman-Turkey Transformation with a random effects model. Further analysis was done with STATA 17.0 statistical software. Additionally, sub-group analysis, publication bias, and sensitivity analysis were performed.

## Result

**Fig. 1:**
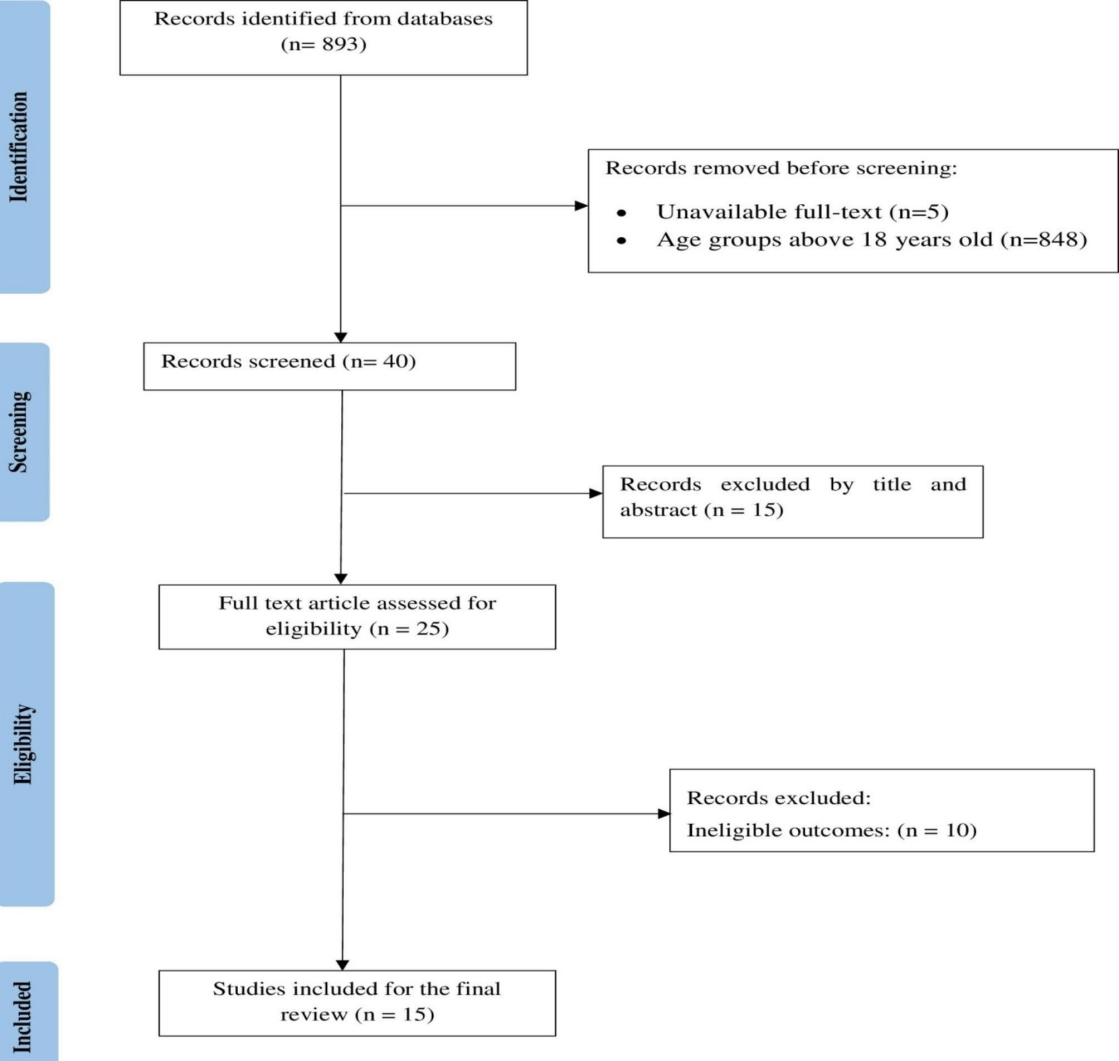
Flow chart illustrating the process of literature search and selection of studies included in the present systematic review and meta-analysis

Finally, 15 studies were included to determine the pooled prevalence of malnutrition in critically ill children (Fig. 1).

Of the 15 included studies, two were in Asia, three in Europe, five in South America, four in North America, and one in Africa. The highest malnutrition prevalence was reported in Africa, Ethiopia (83.5%) [7], whereas the lowest prevalence was reported in Europe, Scotland (13.4%) [14]. Regarding the study design, seven studies were cross-sectional, while eight were cohort studies (Table 1).

**Table 1:** Characteristics of studies included in the systematic review and meta-analysis of malnutrition prevalence among critically ill children

Author [Pub year]	Country	Study design	Sample size	Prevalence (%)
Bagri 2017	India	Cross-sectional	332	51.2
Delgado 2008	USA	Cross-sectional	1077	53
Pollack 1982	USA	Cross-sectional	108	37
Kyle 2013	USA	Cross-sectional	167	18.6
Pollack 1981	USA	Cross-sectional	50	32
De Souza Menezes 2012	Brazil	Cohort	385	45.5
Leite 2013	Brazil	Cohort	221	47.1
Hulst 2004	The Netherlands	Cohort	293	23.9
Feng 2021	China	Cross-sectional	160	41.9
Grippa 2017	Brazil	Cohort	72	81.9
Revuela Iniesta 2019	Scotland	Cohort	82	13.4
Teka 2022	Ethiopia	Cross-sectional	243	83.5
Valla 2019	France	Cohort	579	15
Ventura 2020	Brazil	Cohort	199	18.1
Ventura 2022	Brazil	Cohort	363	22.9

### Prevalence of malnutrition among critically ill children

**Fig. 2:**
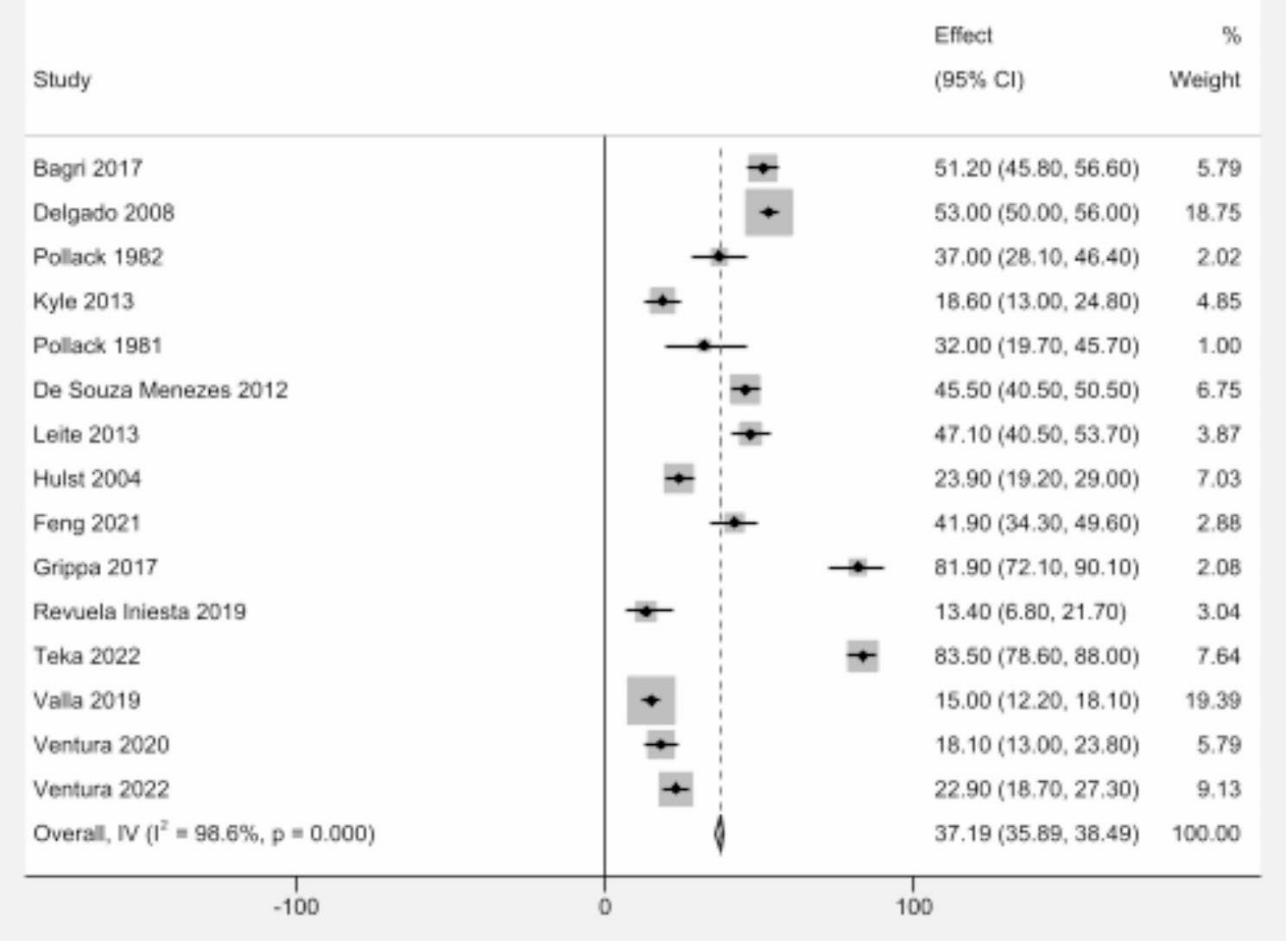
Pooled prevalence of malnutrition among critically ill children

In this meta-analysis, from a total of 15 studies with 4331 study participants, the pooled prevalence of malnutrition in critically ill children was 37.19% (95% CI; 35.89–38.49) with a significant statistical heterogeneity (I^2^ = 98.6, *P* = < 0.0001) (Fig. 2).

### Sub-group analysis

There was a significant heterogeneity among the included studies (I^2^ = 98.6%, *P* = < 0.0001). As a result, a sub-group analysis was done by countries income level, and study design. High income countries reported the lower pooled prevalence of malnutrition among critically ill children (30.14%, 95% CI; 28.41, 31.88). The pooled prevalence of malnutrition was higher in studies with cross-sectional studies (45.6%, 95% CI; 29.7, 62.0) than the cohort studies (Table 2).

**Table 2:** Subgroup analysis of pooled prevalence of malnutrition among critically ill children by level of income, and study design

Variables	Category	No. of studies	Prevalence	95% CI	I-Squared (%)
Income level	Low	1	83.5	78.6–88	0
	Middle	7	38.33	36.17–40.49	97.4
	High	7	30.14	28.41–31.88	98.4
Study design	Cross-sectional	7	52.31	50.33–54.3	98.1
	Cohort	8	25.82	24.1–27.53	97.8
**Pooled prevalence**		**15**	**37.19%**	**35.89–38.49**	**98.6**

### Publication bias and sensitivity analysis

**Fig. 3:**
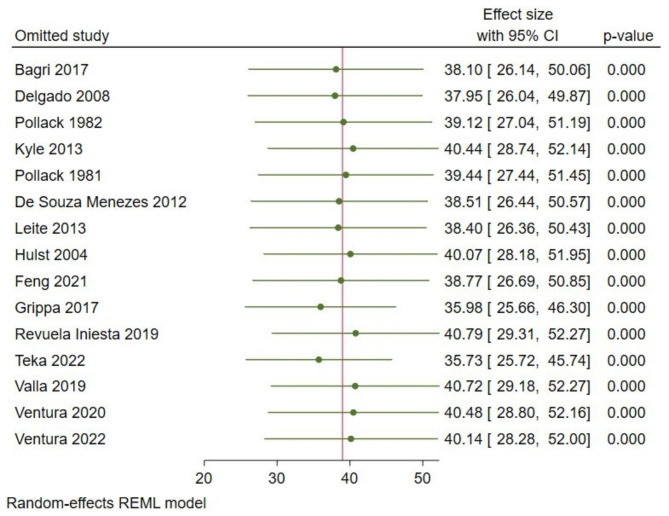
Sensitivity analysis for the study of malnutrition among critically ill children

A sensitivity analysis was performed to evaluate the effect of each study on the pooled estimated prevalence of malnutrition by excluding each study step-by-step with a random effects model. The result showed that the omitted studies did not show a significant difference in the prevalence of malnutrition among critically ill children (Fig. 3).

**Fig. 4:**
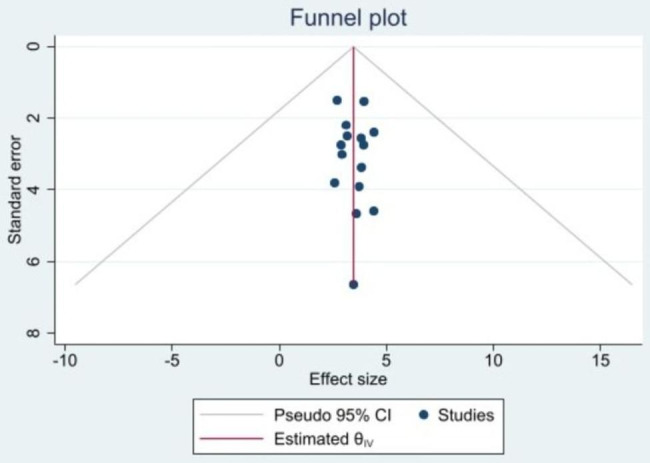
Funnel plot of the prevalence of malnutrition among critically ill children

The included studies were assessed for a potential publication bias with a funnel plot (Fig. 4) and Egger’s test that indicated the absence of a publication bias as *P*-values > 0.05 (Table 3).

**Table 3:** Egger’s test

Std_Eff	Coefficient	Std. err.	t	*P* > t	[95% confidence interval]	
Slope	3.236221	0.4441687	7.29	0.000	2.276652	4.195789
Bias	0.0878343	0.1730347	0.51	0.620	− 0.2859845	0.4616532

## Discussion

This systematic review and meta-analysis reported one primary outcome from the finally retrieved 15 studies aimed to assess the prevalence of malnutrition among critically ill children. The studies were published between 1981 up to 2022 as the studies were not restricted with year of publication.

Of 15 studies among 4331 study participants, the pooled prevalence of malnutrition among critically ill children was 37.19% (95% CI; 35.89–38.49). The finding provides strong evidence of malnutrition among critically ill children, as it revealed that more than one in three critically ill children was malnourished. Factors contributing to the high prevalence might be that critically ill children frequently receive insufficient calorie and protein delivery because enteral or parenteral nutrition cannot be initiated due to gastrointestinal intolerance or the need to restrict fluid intake, initiation is delayed, or there are interruptions in enteral nutrition in order to administer medication or to perform interventions requiring sedation [4]. One of the fundamental roles of nutrition in the healthy child is to enable growth and development. In contrast, the critically ill child uses nutrients principally to defend the body against disease and, even if a high energy delivery is provided, the body is not able to use this for growth. In these children, nutritional treatment must therefore be orientated to delivering those substrates that favor the maintenance of organ function and recovery from disease [15].

In this review, seven studies from the middle income countries reported 38.33% (95% CI; 36.17–40.49) pooled prevalence of malnutrition among critically ill children [14, 16–21]. Despite the fact that malnutrition among children is a major public health problem, particularly in LMIC countries, it also exists in children in high income countries, in families with few resources or due to disturbances of feeding behavior or diseases that affect eating or the absorption of nutrients [22].

In this review, the pooled prevalence of malnutrition was higher in studies with cross-sectional studies with a higher heterogeneity (52.31%, 95% CI; 50.33–54.3; *I*^*2*^ = 98.1%, *p* = < 0.0001) than the cohort studies (25.82%, 95%CI; 24.1–27.53; *I*^*2*^ = 97.8%, *p* = < 0.0001). Cohort studies are more evidence suggestive and the best method for determining the incidence and natural history of a condition [23]. There was no study that showed a significant difference in the prevalence of malnutrition among critically ill children, with no publication bias.

Children who are severely ill frequently suffer from malnutrition, which worsens their prognosis by raising their risk of complications, morbidity, and mortality [22]. Children may experience major medical conditions that require admission to the intensive care unit if undernutrition is not treated with critical care intervention. Therefore, specific methods should be linked with the already-existing healthcare services and nutritional programs to prevent malnutrition among this undeserved group [18]. Children and infants are particularly vulnerable to nutritional issues. Children have lower percentages of muscle and fat than adults, which results in fewer reserves and higher resting energy expenditure. Because of this, children are less tolerant to fasting than adults, they are more vulnerable to protein depletion, and they are more likely to have malnutrition when they experience major illness [22].

It is important to remember that children are still growing and developing, and as such, their nutritional needs are different from those of adults and change depending on their stage of growth. Undernutrition or malnutrition may be a complication of severe illness in children with serious medical issues that necessitate admission to the intensive care unit [24].

### Strength and limitation of the study

More than one reviewer was involved in this systematic review and meta-analysis, and we used a comprehensive search strategy with a JBI critical appraisal instruments as it increased the methodological quality. Besides, both published and unpublished studies were searched to reduce the possibility of selection and publication bias. This review could be the baseline for nutrition program implementers and policymakers to propose possible policies to integrate with the existing health care system based on the prevalence of malnutrition among critically ill children with providing a strong attention. Despite a large scale community based studies has been conducted, this systematic review is the first to be reported as malnutrition in critically ill children: systematic review and meta-analysis. However, the limitations were determinants of malnutrition are not addressed, and articles published other than English language, unavailable full text articles were not included in this review that might be affected the estimation of prevalence of malnutrition. Pooling prevalence and odds ratio despite high heterogeneity is the other drawback of this review. Besides a single study with a high prevalence might be affected the overall prevalence.

## Conclusion

The current systematic review and meta-analysis showed that more than one in three critically ill children was malnourished. Hence, policymakers, program implementers, and nongovernmental organizations are expected to be focus on critically ill children nutritional status. Moreover, further studies should be conducted, particularly, in LMIC.

### Electronic supplementary material

Below is the link to the electronic supplementary material.

Supplementary Material 1

## Data Availability

All relevant data are within the manuscript.
